# Racial differences in time to blood pressure control of aneurysmal subarachnoid hemorrhage patients: A single-institution study

**DOI:** 10.1371/journal.pone.0279769

**Published:** 2023-02-24

**Authors:** Xiaofei Zhou, Adam Hwan Bates, Uma V. Mahajan, Ansh Desai, Jeffrey Butke, Berje Shammassian, Yifei Duan, Christopher Burant, Kaylee Sarna, Martha Sajatovic, Dhimant Dani, S. Alan Hoffer

**Affiliations:** 1 Department of Neurological Surgery, University Hospitals Cleveland Medical Center, Cleveland, OH, United States of America; 2 Cerebrovascular Center, Cleveland Clinic, Cleveland, OH, United States of America; 3 School of Medicine, Case Western Reserve University, Cleveland, OH, United States of America; 4 Division of Neurocritical Care, Department of Neurology, University of Miami Miller School of Medicine, Miami, FL, United States of America; 5 Frances Payne Bolton School of Nursing, Case Western Reserve University, Cleveland, OH, United States of America; 6 Behavioral Health Research Group, Case Western Reserve University, Cleveland, OH, United States of America; 7 Neurological and Behavioral Outcomes Center, University Hospitals Cleveland Medical Center, Cleveland, OH, United States of America; Bayero University Kano, NIGERIA

## Abstract

**Background and purpose:**

Aneurysmal subarachnoid hemorrhage occurs in approximately 30,000 patients annually in the United States. Uncontrolled blood pressure is a major risk factor for aneurysmal subarachnoid hemorrhage. Clinical guidelines recommend maintaining blood pressure control until definitive aneurysm securement occurs. It is unknown whether racial differences exist regarding blood pressure control and outcomes (HLOS, discharge disposition) in aneurysmal subarachnoid hemorrhage. Here, we aim to assess whether racial differences exist in 1) presentation, 2) clinical course, and 3) outcomes, including time to blood pressure stabilization, for aSAH patients at a large tertiary care medical center.

**Methods:**

We conducted a retrospective review of adult aneurysmal subarachnoid hemorrhage cases from 2013 to 2019 at a single large tertiary medical center. Data extracted from the medical record included sex, age, race, insurance status, aneurysm location, aneurysm treatment, initial systolic and diastolic blood pressure, Hunt Hess grade, modified Fisher score, time to blood pressure control (defined as time in minutes from first blood pressure measurement to the first of three consecutive systolic blood pressure measurements under 140mmHg), hospital length of stay, and final discharge disposition.

**Results:**

194 patients met inclusion criteria; 140 (72%) White and 54 (28%) Black. While White patients were more likely than Black patients to be privately insured (62.1% versus 33.3%, p < 0.001), Black patients were more likely than White patients to have Medicaid (55.6% versus 15.0%, p < 0.001). Compared to White patients, Black patients presented with a higher median systolic (165 mmHg versus 148 mmHg, p = 0.004) and diastolic (93 mmHg versus 84 mmHg, p = 0.02) blood pressure. Black patients had a longer median time to blood pressure control than White patients (200 minutes versus 90 minutes, p = 0.001). Black patients had a shorter median hospital length of stay than White patients (15 days versus 18 days, p < 0.031). There was a small but statistically significant difference in modified Fisher score between black and white patients (3.48 versus 3.17, p = 0.04).There were no significant racial differences present in sex, Hunt Hess grade, discharge disposition, complications, or need for further interventions.

**Conclusion:**

Black race was associated with higher blood pressure at presentation, longer time to blood pressure control, but shorter hospital length of stay. No racial differences were present in aneurysmal subarachnoid hemorrhage associated complications or interventions.

## Introduction

Aneurysmal subarachnoid hemorrhage (aSAH) occurs in approximately 30,000 patients every year in the United States and has an overall global incidence of 7.9 per 100,000 person-years [1, 2]. Pre-hospital mortality for aSAH has been estimated to be up to 25%, with in-hospital mortality in recent studies estimated to be approximately 14% [3, 4]. Of those who survive initial rupture, many still face the risks of rebleeding, acute hydrocephalus, vasospasm and delayed cerebral ischemia. Prior to aneurysm securement, the risk of rebleeding has been estimated to range between 4 and 14%”within the first 24 hours, with maximum risk in the first 2 to 12 hours [5].

Blood pressure control after presentation is thought to impact rebleeding risk. The American Stroke Association’s 2012 guidelines recommend maintaining blood pressure (BP) below 160 mmHg as a means of mitigating re-rupture risk prior to aneurysm securement [6]. Blood pressure control is also important as a chronic disease factor, with uncontrolled hypertension among the most significant risk factors for aSAH and also linked to worse outcomes [7–12]. Yet, it remains unclear how uncontrolled chronic hypertension affects timing to blood pressure control following subarachnoid hemorrhage, and therefore, complications such as re-rupture.

Black Americans have a significantly higher prevalence of both hypertension and uncontrolled hypertension compared with their White counterparts [13]. Mortality rates for aSAH are higher for Black compared to White Americans [14, 15]. It is unclear if this is due to differences in treatment or underlying comorbidities. It is also currently unknown whether there are racial differences in clinical course of BP control in aSAH patients.

Here, we aim to assess whether racial differences exist in presentation, clinical course, and outcomes (HLOS, disposition, complications, need for interventions, time to blood pressure stabilization) for aSAH patients at a large tertiary care medical center.

## Methods

Approval was obtained from University Hospitals Cleveland Medical Center (UHCMC) Institutional Review Board, and the need for consent was waived for this study (IRB number 20191604). We conducted a single-institution retrospective review of all aSAH cases between 2013 and 2019 at a large tertiary medical center, UHCMC. Inclusion criteria were adult patients (age ≥ 18); aSAH as seen on computed tomography of the head or computed tomography angiography, digital subtraction angiography, or magnetic resonance imaging; saccular aneurysms; dissecting aneurysms; and patient arrival to our center within 48 hours of ictus. Exclusion criteria were prior SAH; withdrawal of care prior to aneurysm treatment; absence of aneurysm on imaging (e.g., non-aneurysmal perimesencephalic bleed, traumatic SAH) or incidental aneurysm finding unrelated to current admission; mycotic aneurysm; poor baseline functional status (baseline modified Rankin Scale [mRS] > 2); delayed presentation (arrival to our hospital more than 48 hours after ictus); aneurysm associated with arteriovenous malformation; and elective aneurysm clipping (non-ruptured aneurysm).

Medical records were reviewed to collect the relevant information. Demographic data including age, sex, comorbidities, and race were identified from medical records. Clinical information included location of initial patient presentation (UHCMC, affiliated community hospital, or outside hospital); aneurysm location (anterior circulation or posterior circulation); initial systolic blood pressure (SBP); initial diastolic blood pressure (DBP); Hunt Hess grade (grades 1–5); Modified Fisher score (graded 1–4); placement of an external ventricular drain (EVD) prior to final securement; presence of rebleed; securement method (clipping or coiling); occurrence of vasospasm requiring treatment (either vasopressors, angioplasty, intraarterial verapamil, or intrathecal nicardipine); presence of Takotsubo stress cardiomyopathy (defined as reduced ejection fraction with apical ballooning on echocardiogram); mRS on discharge; whether a tracheostomy, percutaneous endoscopic gastrostomy (PEG), or ventriculoperitoneal shunt were placed during admission; time (in minutes) to blood pressure control, and final discharge disposition.

Time to blood pressure control was defined as the time from first blood pressure measurement to the first of three consecutive systolic blood pressure measurements under 140 mmHg per institutional practice. The first recorded blood pressure measurement was used regardless of location of read (i.e. readings at outside hospitals or ambulances were included). Discharge disposition was categorized as good (home with no needs, home with home care, or rehabilitation) or poor (skilled nursing facility, long term acute care hospitals, or hospice/deceased). We collected past medical history data on the following comorbidities: congestive heart failure, type 2 diabetes mellitus, hypertension, hyperlipidemia, coronary artery disease (with or without stent), prior myocardial infarction, connective tissue disorder, end stage renal disease, current or prior alcohol abuse, current or prior smoking, coagulopathy or use of anticoagulants/antiplatelets, atrial fibrillation, deep vein thrombosis or pulmonary embolism, liver dysfunction, and bleeding dyscrasia.

Stata 15 (StataCorp. 2019. College Station, Tx) software was used for statistical analysis. Due to small numbers, patients whose race was not White or Black were not included in statistical analyses. As race was dichotomous, Chi square analyses were conducted for categorical outcomes. Independent samples t-tests were run for continuous outcomes. Median tests, a nonparametric equivalence to the t-test, were run when the continuous outcomes were not normally distributed. Linear and logistic regression tests were performed, for continuous and dichotomous outcomes respectively, to control for patient age, sex, initial diastolic and systolic blood pressures, and modified Fisher scores.

## Results

Patient demographics and presentation characteristics are displayed in Table 1 while their clinical management and outcomes are displayed in Table 2.

**Table 1:** Demographics and presentation of included patients.

	White (n = 140)	Black (n = 54)	p-value
**Sex n (%)**			**0.209**
**M**ale	41 (29.3%)	11 (20.4%)	
**F**emale	99 (70.7%)	43 (79.6%)	
**Age mean (sd)**	59.25 (13.44)	52.92 (14.36)	**0.004**
**Institution at arrival n (%)**			**<0.001**
Outside Hospital	61 (43.6%)	8 (14.8%)	
**C**ommunity hospital	58 (41.4%)	15 (27.8)	
**Study Site**	21 (15.0%)	31 (57.4%)	
**Insurance type n (%)** *			
Private	87 (62.1%)	18 (33.3%)	**<0.001**
Medicare	57 (40.7%)	12 (24.1%)	**0.032**
Medicaid	21 (15.0%)	30 (55.6%)	**<0.001**
**Number of comorbidities mean (sd)**	1.96 (1.48)	1.70 (1.25)	**0.268**
**Aneurysm location n (%)**			
Anterior	94 (67.1%)	36 (66.7%)	**0.95**
Posterior	52 (37.1%)	21 (38.9%)	**0.822**
Both	6 (4.3%)	3 (5.6%)	**0.706**
**EVD placement n (%)**			**0.465**
**Y**es	73 (52.1%)	25 (46.3%)	
**N**o	67 (47.9%)	29 (53.7%)	
**Aneurysm treatment n (%)**			**0.27**
Clip	68 (48.6%)	31 (57.4%)	
Coil	72 (51.4%)	23 (42.6%)	
**Aneurysm grade mean(sd)**			
Hunt Hess	2.46 (1.11)	2.30 (1.24)	**0.361**
Modified Fisher	3.48 (.87)	3.17 (1.11)	**0.04**
**Blood pressure (median)**			
Systolic Blood Pressure (mmHg)	148	165	**0.004**
Diastolic Blood Pressure (mmHg)	84	93	**0.02**

*Percentages total over 100% because some patients had multiple insurances.

**Table 2:** Management and clinical outcomes.

	White (n = 140)	Black (n = 54)	p-value
**Time to BP control (minutes—median)**	90	200	**0.001**
**Hospital Length of Stay (days—median)**	18	15	**0.031**
Male	18	16	**0.308**
Female	18	15	**0.058**
**Disposition n (%)**			**0.808**
Good (home, home w/ home health, rehab)	96 (68.6%)	38 (70.4%)	
Poor (SNF, LTACH, hospice/death)	44 (23.5%)	16 (29.7%)	
**Complications n (%)**			
Rebleed			**0.898**
Yes	3 (2.1%)	1 (1.9%)	
No	137 (97.9%)	53 (98.1%)	
Vasospasm requiring treatment			**0.419**
Yes	36 (25.7%)	17 (31.5%)	
No	104 (74.3%)	37 (68.5%)	
Takatsubo’s			**0.456**
Yes	9 (6.5%)	2 (3.7%)	
No	130 (93.5%)	52 (96.3%)	
**Need for interventions**			
Presence of tracheostomy	16 (11.4%)	5 (9.3%)	**0.663**
Presence of Percutaneous Endoscopic Gastrostomy Tube	41 (29.3%)	9 (16.7%)	**0.072**
Presence of shunt	25 (17.9%)	9 (16.7%)	**0.845**

*SNF: Skilled Nursing Facility; LTACH: Long Term Acute Care Hospital.

### Patient demographics

Of the 546 patients identified in the aSAH database, 200 met clinical inclusion criteria. Six patients, whose racial classification was not White or Black, were not included in statistical analysis because this group was underpowered to analyze separately. 194 patients were included for statistical analysis. 140 were White patients and 54 were Black patients. The mean age was 59.25 ± 3.44 years for White patients and 52.92 ± 14.36 years for Black patients (p = 0.004). 70.7% of White patients and 79.6% of Black patients were female (p = 0.209). White patients initially presented to an outside hospital or community hospital 85.0% of the time, while Black patients presented directly to the tertiary care center in the majority of cases (57.4%, p < 0.001). A greater proportion of White patients compared to Black patients were privately insured (62.1% versus 33.3%, p < 0.001).

There were more White patients compared to Black patients with Medicare insurance (40.7% versus 24.1%, p = 0.032). whereas a larger proportion of Black patients had Medicaid in comparison to their White counterparts (55.6% versus 15.0%, p < 0.001). There was not a significant difference in the number of comorbidities at presentation between White and Black patients (p = 0.268).

### Acute subarachnoid hemorrhage characteristics

Race was not significantly associated with aneurysm location or Hunt Hess score. However, Black patients had mean lower modified Fisher Score (3.17 ± 1.11 versus 3.48 ± 0.87 for White race, p = 0.04). No significant racial difference was present in the aneurysm treatment approach (48.6% of White patients underwent clipping versus 57.4% of Black patients, p = 0.27) or EVD placement frequency (p = 0.465).

### Blood pressure characteristics

Black patients presented with a median SBP of 165 mmHg and White patients presented with a median SBP of 148 mmHg (p = 0.004). The median DBP on presentation was 93 mmHg for Black patients and 84 mmHg for White patients (p = 0.02). The median time to blood pressure control was 200 minutes after initial presentation for Black patients, compared to 90 minutes after initial presentation for White patients (p = 0.001).

### Clinical outcomes

White patients had a median hospital length of stay (HLOS) of 18 days and Black patients had a median HLOS of 15 days (p < 0.031). Discharge disposition was not significantly different between White and Black patients (p = 0.808). Race was not associated with rebleed risk (p = 0.898), vasospasm incidence (p = 0.419), or Takotsubo cardiomyopathy incidence (p = 0.456). The placement of tracheostomy, PEG, or shunt was not significantly different between White and Black patients.

### Adjusted outcomes

Linear regression of time to BP control adjusted for patient race, age, sex, initial DBP and SBP, modified Fisher scores, and insurance type demonstrated that Black race (p = 0.010), higher initial SBP (p = 0.002), and lower modified Fischer score (p = 0.019) were significant predictors of longer time to blood pressure control. Linear regression of hospital length of stay adjusted for the same variables demonstrated that a higher Modified Fischer score was a significant predictor of longer stay (p < 0.001).

## Discussion

In this study, compared to White aSAH patients, Black aSAH patients presented with higher initial SBP and DBP, and had a longer time to BP control. Black patients continued to demonstrate a longer time to BP control even when adjusted for patient age, sex, initial SBP and DBP, and modified Fisher scores. A variety of factors may underlie these racial differences.

The mean age for Black patients was roughly seven years younger than White patients, a statistically significant difference. Despite the younger age, black patients had higher SBP and DBP at initial presentation, suggesting that Black patients may have poorer underlying health. This is associated with a myriad of factors, potentially including worse access to preventative medical care, chronic stress, financial status, or environmental exposures [16]. Our findings are in concordance with past work in aSAH patients that also demonstrated younger age and higher rate of hypertension with associated elevated blood pressure at the time of admission in Black patients relative to White patients [15]. BP at initial presentation has been shown to impact SAH complications, specifically rebleed risk [17, 18]. However, in the present study, Black and White patients showed no difference in rebleed or vasospasm risk, incidence of Takotsubo cardiomyopathy, or need for post-rupture interventions (e.g., placement of PEG, shunt, or tracheostomy) despite differences in BP at presentation. Regardless, the difference in age of presentation is concerning; aSAH can have drastic impacts on quality of life and employment in the short and long term [19–21]. While the rate of discharge to skilled nursing facilities and long-term care facilities was not significantly different between White and Black patients, an earlier onset of disability or reduction in employment could contribute to a higher lifetime burden of disease.

The increased prevalence of HTN among Black patients has been established in previous studies and is thought to be multifactorial, with socioeconomic status, social support, and cultural experiences serving as possible contributing factors [22–24]. Specifically, lower annual income and lack of healthy physician-patient relationships that could foster lifestyle modifications are thought to be two of the central driving factors for the increased prevalence of HTN among Black patients [22, 24]. In addition to differences in risk factors for hypertension, hypertension itself may be managed differently between racial groups in the outpatient setting, specifically in terms of medications employed [25]. Regardless of the cause, the difference in BP at presentation is concerning because hypertension may increase the risk of post-SAH complications, such as postoperative seizures [26]. Furthermore, poorer control of HTN at baseline may partly explain why Black patients had a significantly longer time to BP control in this study. This is noteworthy, given that increased time spent outside the limits of cerebral autoregulation is correlated to higher 90-day mRS scores [27].

Uncontrolled blood pressure is a factor that has been historically associated with aSAH incidence and outcomes [5, 17, 18, 28]. A prior study in Japan suggested that high initial systolic blood pressure (≥220 mmHg) is a risk factor for higher grade hemorrhage [29]. One study with 612 aSAH patients, all with BP controlled to 120–160 mmHg, found that increased BP variability correlated with a significantly increased risk of rebleed [30]. A meta-analysis of 14 studies and 5693 patients found elevated BP (either greater than 140 mmHg or 160 mmHg, depending on institutional practice) was associated with double the risk of rebleed compared to normotension [17]. The optimal magnitude of blood pressure reduction has not been well established, and there remains substantial practice variability following aSAH [31]. Regardless, rebleeds are associated with drastic loss of functional independence [32]. Therefore, it is important to investigate the role of BP management in aSAH outcome and complications rates, especially in light of racial disparities in blood pressure management in the inpatient acute and outpatient chronic settings.

Although there were significant differences in SBP, DBP and time to BP control between White and Black patients, this did not translate to differences in rates of complications or disposition at discharge. This is surprising in the context of current clinical guidelines and earlier data, which suggest that elevated BP is correlated with worse outcomes [5, 17, 18, 28]. It is possible that the time to blood pressure control in our institution is below the clinically relevant length of time, perhaps contributing to the lack of correlation between outcomes and longer length of time to control seen in our study. Furthermore, given the relatively rare occurrence of rebleed, the current study may not be powered to identify a significant difference in the risk of rebleed between races.

Paradoxically, Black patients had a shorter HLOS and lower mean modified Fisher Scores than White patients despite worse BP control. A shorter HLOS among Black patients is surprising given that numerous studies have found longer HLOS for Black patients treated both by neurosurgery and by other specialties [33–38]. One possible explanation for the shorter HLOS may be that in our study, Black patients were significantly more likely to present at UHCMC, the site of final aneurysm securement. This could have led to earlier neurosurgical consultation and definitive management, which has been found to produce better outcomes, including reduced HLOS [39, 40].

Insurance status previously has also been shown to impact HLOS. Specifically, past literature has found uninsured patients have shorter HLOS whereas patients with Medicaid/Medicare have longer HLOS [41, 42]. There are well established disparities in insurance rates between White and Black Americans, with Black Americans being far more likely to be uninsured (15.9–10.1% vs 9.8–6.3%) [43, 44]. This study found that White patients were more likely than Black patients to have private insurance or Medicare and less likely to have Medicaid. However, adjusted regression demonstrated that insurance type was not a significant predictor of HLOS.

Similarly, the significantly lower modified Fisher score in Black patients was unexpected. Modified Fisher score has been increasingly validated as a predictor of outcome, including delayed cerebral ischemia, cognitive function, functional outcome, and death [45–48]. Subarachnoid hemorrhage severity, as represented by the modified Fisher score, has been found to be associated with age, hypertension, and elevated SBP on admission [49]. In this context, one would have expected the higher average SBP, DBP, and rates of hypertension in Black patients to be correlated with a higher Fisher score. This, however, was not what was observed in our population. It is possible that the lower age seen in Black patients may have served as a significant protective factor. Additionally, although the difference in modified Fisher score between races was statistically significant, it may not have been a clinically meaningful difference given the small magnitude and poor interrater reliability of the scale [50].

Our study is limited in several respects, including its single-site nature. We evaluated a single metropolitan area, which may in turn influence determinants of health unique to the included patient demographic. Ultimately, multicenter research is needed to validate these findings and better elucidate how differences in blood pressure and blood control times may impact clinical and functional outcomes. In addition, racial data was extracted from the medical record rather than self-reported by patient. Furthermore, this study only considered Black and White patients, as there was an insufficient number of patients of other races to perform statistical analysis. Such racial data could have presented further nuances to the analysis. Long-term outcomes following discharge were also unavailable. Therefore, there may have been racial disparities regarding long-term recovery and functional outcomes, or readmissions and follow-up care that are unobserved [22–24, 51]. Additionally, BP control data was determined based on routine BP collection times, so the absolute time to control may not be represented in the data set and minutes to BP control may be overestimated. Lastly, although we included seven years of patient data, with a starting date coinciding with the hospital adoption of an electronic medical record and thus capturing all available data, our study only included 194 patients and thus may have been underpowered to detect additional differences.

## Conclusions

We found that Black race was associated with higher BP at presentation and longer time to BP control, as compared to White race. These racial differences were not associated with an increase in complications or in-hospital interventions. Although this study did not demonstrate significant racial differences in terms of disposition as primary outcome, there does exist significant differences in initial blood pressure and time to control. These racial differences, however, may contribute to differences in outcome, such as long term health measures, that were not measured by this study. Therefore, further investigation is warranted and necessary.
